# Novel electrostatic dry lift-off and transfer route for vertically aligned nanocomposite oxide thin films

**DOI:** 10.1186/s40580-025-00494-1

**Published:** 2025-07-18

**Authors:** Matthew P. Wells, Babak Bakhit, Simon M. Fairclough, Jordi J. H. Weingard, Caterina Ducati, Judith L. MacManus-Driscoll

**Affiliations:** 1 Department of Materials Science and Metallurgy, University of Cambridge, 27 Charles Babbage Road, Cambridge, CB3 0FS UK; 2 Thin Film Physics Division, Department of Physics (IFM), Linköping University, 58183 Linköping, Sweden

## Abstract

Highly oriented oxide thin films hold substantial relevance to a wide range of fields. A major challenge is their integration with technological substrates, such as flexible polymers and silicon. While multiple strategies for the lift-off and transfer of high-quality oxide thin films have been widely explored, it remains a challenge to easily transfer films with low defect levels. In this work, we introduce a novel and effective strategy for achieving high-quality, freestanding perovskite oxide thin films. We first demonstrate that highly oriented perovskite oxides, as both single-phase films and vertically aligned nanocomposite (VAN) films, can be grown by pulsed laser deposition on single crystal NaCl, as not shown before. We next show that the VAN films, unlike single-phase films, can be readily, electrostatically, dry lifted-off the substrate. The success of the lift-off technique is enabled by (i) a high thermal expansion mismatch of the film, producing compression in the film, and (ii) lack of elastic strain relief in the out-of-plane direction in the VAN film. Finally, we show that a VAN cathode film can be incorporated into a proof-of-concept micro-solid oxide fuel cell structure, and that it is of good structural quality as demonstrated by performance comparable to equivalent VAN films grown on single crystal YSZ. Thus, we developed an entirely new way to lift-off and transfer highly oriented oxide thin films for use in a wide variety of electronic applications.

**Graphical Abstract:**

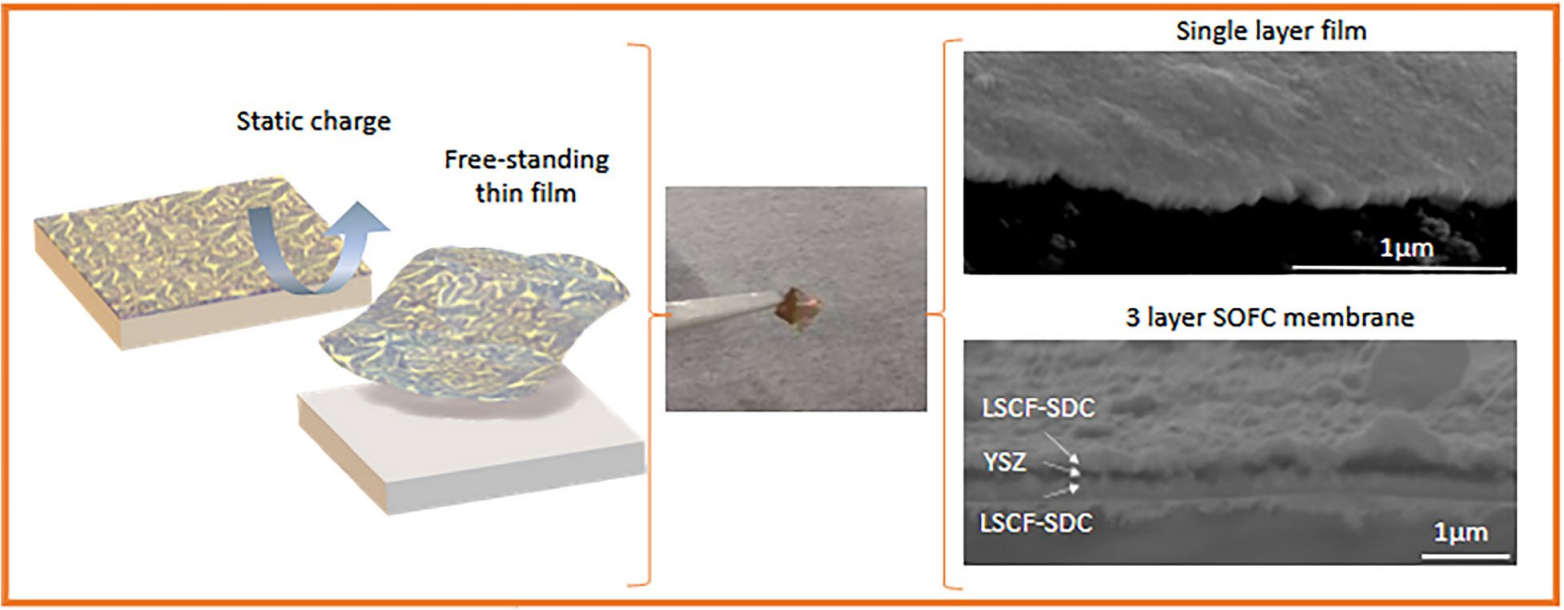

**Supplementary Information:**

The online version contains supplementary material available at 10.1186/s40580-025-00494-1.

## Introduction

Multiple strategies have been explored for achieving freestanding thin films, and their transfer to technologically relevant substrates such as flexible polymers and silicon. Proposed strategies include remote epitaxy [1, 2], sacrificial buffer layers [3], soluble substrates [4], and mechanical exfoliation [5]. For epitaxial oxide films, the sacrificial buffer layer approach, in which epitaxial water-soluble buffer layers are grown on single crystal substrates by physical vapour deposition, is most popular [6–9]. A recent work even demonstrates the possibility to grow high-quality epitaxial La_0.7_Sr_0.3_MnO_3_ films grown by pulsed laser deposition (PLD) on a solution deposited Ca-doped Sr_3_Al_2_O_6_ buffered SrTiO_3_ substrate [10]. However, the above approaches all suffer different levels of deleterious buckling and/or cracking.

A simple lift-off method which mitigates crack formation and does not rely on the need for expensive, area-limited single crystal substrates is desired. One such approach is the use of water-soluble single crystal NaCl substrates, which are more cost-effective and can be grown in cm^2^ area [11]. While several materials of different crystal structures such as anatase TiO_2_, Au, Cu_2_O and halide perovskite such as CsSnBr_3_ have been grown epitaxially [12–15], to the best of our knowledge no studies have explored the deposition of perovskite oxides on NaCl. Nevertheless, these works highlight that highly oriented growths can indeed take place for different structures on the rock salt cubic NaCl, despite mismatched lattice parameters. However, dissolution of the substrate has the disadvantages of potential chemical damage (e.g. hydroxide formation) to the oxide films, and mechanical damage when transferring the film from the water to another substrate [16].

In this work, we implement a simple, dry, electrostatic lift-off and transfer method and, in so doing, remove the requirement of water dissolution of the substrate. Our novel transfer strategy is specific to vertically aligned nanocomposite (VAN) films grown on single crystal NaCl substrates. A key advantage of this strategy is the avoidance of the need for additional experimental steps to achieve film lift-off, for instance the deposition of graphene, application of structural supports, and application and removal of thermal release tapes as required by remote epitaxy techniques [1, 17]. In the present work, we show that highly oriented VAN films can instead be lifted directly from the NaCl substrate and placed on the substrate of choice.

VAN films offer unique functionalities and advantages over their planar counterparts and have therefore emerged as a leading class of materials in a very wide range of fields [18] e.g. improved, ionic, ferroelectric, magnetoelectric, photoelectrochemical, photocatalytic, catalytic, resistive switching properties [19–26]. They can be of two forms, either epitaxial [20, 21] or highly oriented (i.e. oriented out-of- plane, but not in-plane [26, 27]). The benefit of epitaxial VANs is a lack of grain boundaries and so reduced scattering of carriers, along with fewer defects within grains due to high growth temperatures. Meanwhile, the benefits of oriented VANs include lower temperature growth, and lack of need for expensive, small single crystal substrates. While oriented VANs may exhibit lower crystalline perfection and higher grain boundary/defect concentrations, this is not detrimental to some functional effects where surface reactions are dominant. Indeed, we have shown this to be the case for solid oxide cathode systems [26, 27]. More work is needed to explore other surface dominated effects which could benefit from VANs, e.g. photocatalysis.

Herein, we explore the role of interfacial mismatch strain between film and substrate as well as the role of the film structure (VAN versus planar) on the success of the lift-off process. We find that the dry lift-off process is effective for VAN films though not for single-phase planar films. We demonstrate this for 3 different VAN film compositions where there is a compressive strain between the film and the NaCl single crystal substrate upon cooling, which arises due to their thermal expansion coefficient (CTE) mismatch. The particular nature of the strain state in VAN films gives rise to film buckling, which in turn readily enables lift-off using an electrostatic force.

The applicability of the dry lift-off process is demonstrated using a (La_0.60_Sr_0.40_)_0.95_Co_0.20_Fe_0.80_O_3_-(Sm_2_O_3_)_0.20_(CeO_2_)_0.80_ (LSCF-SDC) VAN film composition. This is an excellent model composition to prove the value and effectiveness of the dry lift-off process, as such nanocomposites are excellent cathode materials in micro solid oxide cells (µSOC) [27–29]. Hence, the lift-off transfer method is a potential route for incorporating thin films into µSOCs, while circumventing the complex microfabrication processing demanded by direct growth on Si [30–32]. To perform well, the cathode film must be unfractured and not degraded chemically; hence, the demonstration of good cathode performance of a lifted-off and transferred LSCF-SDC VAN film serves as proof that the dry lift-off process is effective to provide high structural and chemical film quality [30].

Herein, we show that an LSCF-SDC VAN film lifted off and transferred in this manner can indeed be implemented in a proof-of-concept symmetric fuel cell membrane with 5 × 5 mm area and excellent electrochemical performance. Thus, the present work demonstrates the successful implementation of a novel dry, electrostatic lift-off transfer process of oxide thin films, which have applicability in a wide variety of fields.

## Results and discussion

**Fig. 1:**
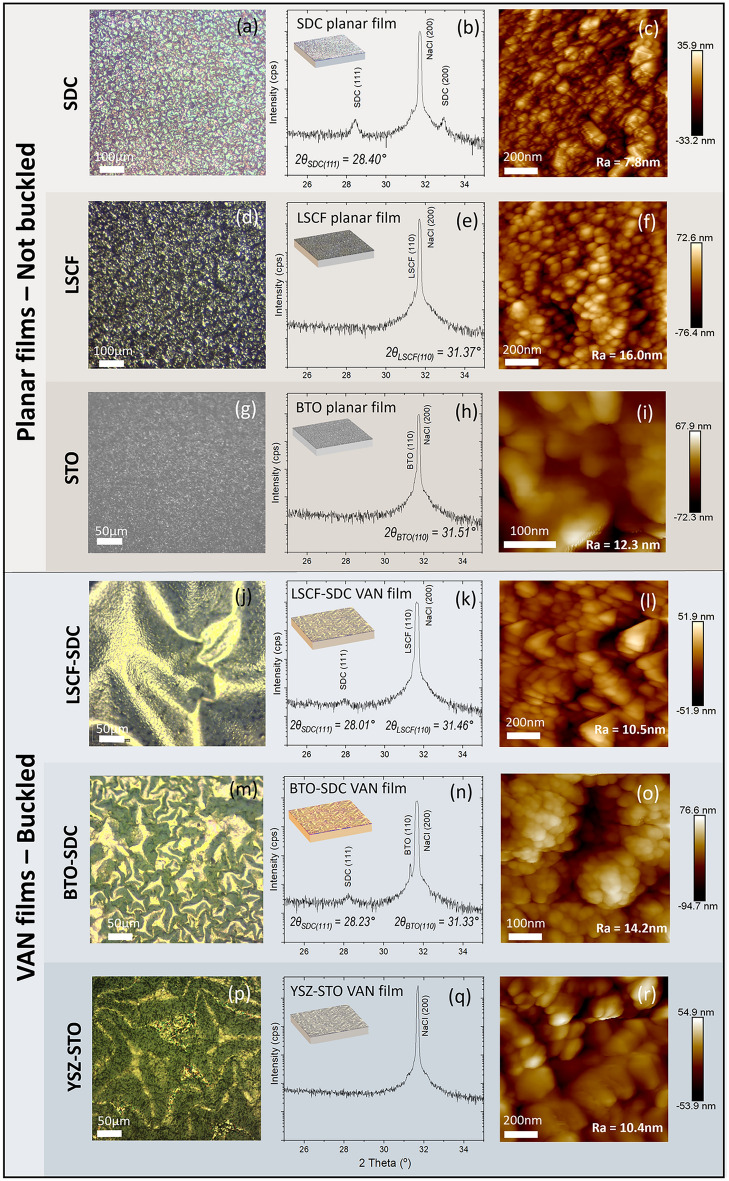
Optical images, XRD scans and AFM images (from left to right) of the different films grown on NaCl of this study. **a**–**c** SDC films; **d**–**f** LSCF films; **g**–**j** BTO films **j**–**l** LSCF-SDC VAN films; **m**–**o** BTO-SDC VAN films; **p**–**r** YSZ-STO VAN films. Here XRD plots in **k** and **n** show highly oriented growth of SDC as part of the VAN films. Optical microscopy images show that buckle delamination is unique to the VAN films. AFM images show that the surface roughness of the buckled VAN films is not significantly increased compared to the planar counterpart films

**Fig. 2:**
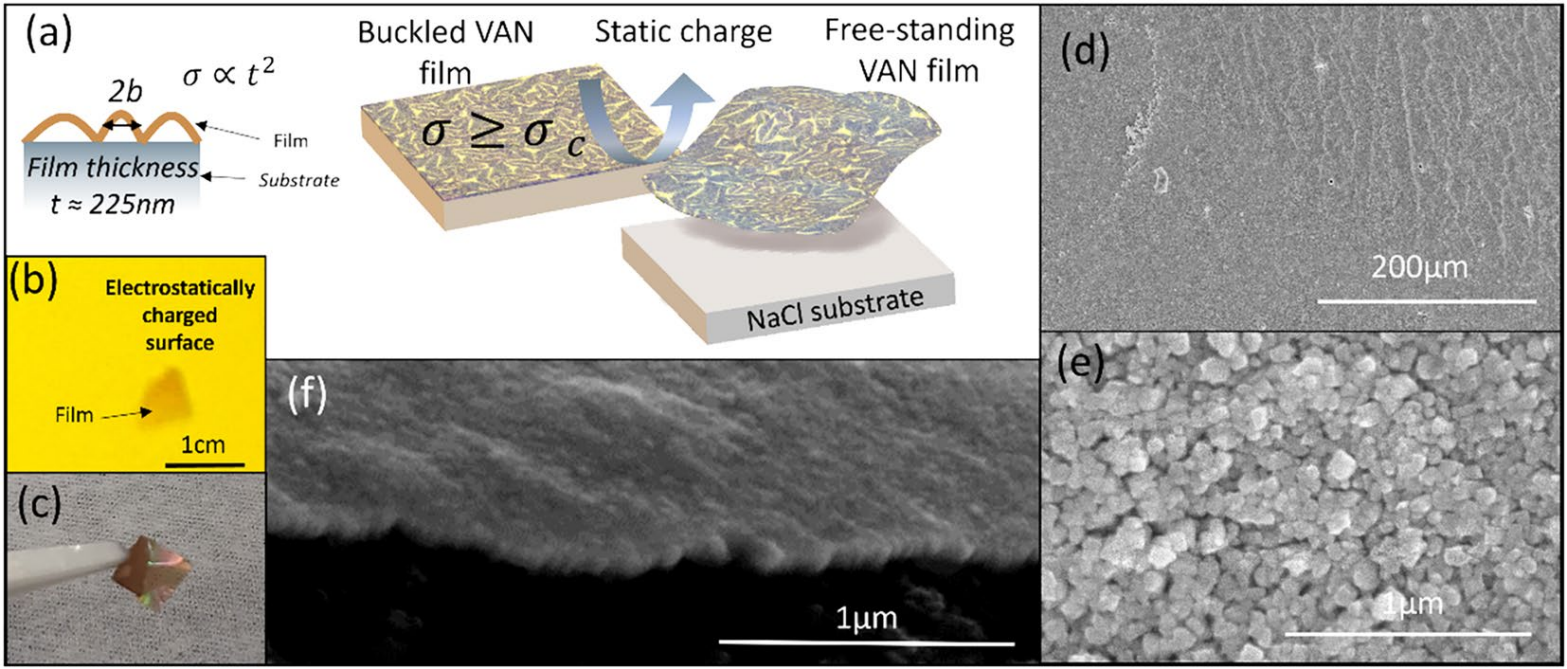
**a** Schematic illustrations of buckle delamination in the VAN films of this study with thickness t, buckle half-width b, and critical stress σ_c_; **b** Photograph of film transferred to electrostatically charged rubber surface; **c** Photograph of freestanding LSCF-SDC VAN film with 5 mm x 5 mm area; **d** top-down SEM showing crack- free transferred VAN film; **e** higher magnification top-down SEM of transferred VAN film; **f** Cross-sectional SEM of freestanding VAN film

Six different film compositions were studied, including three single phase planar films ((Sm_2_O_3_)_0.20_(CeO_2_)_0.80_ (SDC), (La_0.60_Sr_0.40_)_0.95_Co_0.20_Fe_0.80_O_3_ (LSCF), and BaTiO_3_ (BTO)) and three VAN films (LSCF-SDC, BTO-SDC and (Y_2_O_3_)_0.08_(ZrO_2_)_0.92_–SrTiO_3_ (YSZ-STO)). Typically, such VAN films are characterised by a self-assembled nanostructure, in which nanopillars of one material are embedded in a matrix of another [33–35]. Figure 1a, d, g, j, m, p show optical microscopy images of each film grown to a thickness of ~ 225 nm by PLD on NaCl substrates at 590 °C and cooled to room temperature. Here, we observe a buckle delamination of the film from the substrate which occurs *exclusively* in the VAN films and *not* the single-phase planar films. Figure 1 also compares XRD patterns near the main NaCl(200) peak, and AFM-obtained surface micrographs of the different planar single phase and VAN films of this study. It is immediately visually apparent that all the films show few XRD peaks, indicative of strong film orientation, and that the planar single-phase films are relatively flat compared to the buckled appearance of the VAN films. Full XRD scans for all samples are shown in Fig. S1. Fig. S2 shows the optical surface topography of an LSCF-SDC VAN film grown to a thickness of ~ 50 nm that exhibits the same surface wrinkling, albeit with a reduced buckle half width* b* (as shown in Fig. 2a) relative to the ~ 225 nm thick film. This is consistent with Eq. 1 relating critical stress (σ_c_) in the film-to-film thickness (*t*) and buckle half width* b* [36]. (*E*_*f*_ denotes the Young’s modulus, *v*_f_ the Poisson ratio). The equation can be readily rearranged to show proportionality between *t* and* b*.

1 $${\sigma }_{c}=\frac{{\pi }^{2}{E}_{f}}{12(1-{v}_{f}^{2})}{\left(\frac{t}{b}\right)}^{2}$$

Thus, buckle delaminations form to release stored elastic energy, well-documented in compressively strained films [36–38]. In this case, the delamination allows us to develop the film transfer strategy as outlined schematically in Fig. 2a. After application of a small force (< ~ 1 N) from an electrostatically charged rubber surface, the film is lifted off. As an example, Fig. 2b shows a photograph of the film transferred onto the surface of a rubber balloon. Thus dry, chemical-free transfer to an arbitrary substrate is achieved. We note here that the films can be transferred either directly to a desired electrostatically charged substrate, or a charged surface can be used as an intermediate substrate prior to transfer to a new substrate, followed by the re-use of the original NaCl substrate. As demonstrated in several previous works, Van der Waals forces are sufficient for adhesion of the freestanding film to a new substrate [39–41]. For applications where greater adhesion strength is required, complimentary techniques may be considered, for example, the use of epoxy adhesives as demonstrated in the adhesion of GaN films to Si [42].

Film delamination and buckling has previously been explored in some detail in a range of different film materials [43, 44]. In single phase films, it has been shown that as film thickness (*t*) increases, the intrinsic stress of the film (*σ*) increases (*σ* µ *t*^2^) [36] until it exceeds the critical compressive biaxial stress (σ_c_) leading to the onset of buckle delamination. For practical applications, it is crucial that buckled films do not crack. Indeed, the films shown in Fig. 2 do not crack upon buckling, and this can be attributed to the fact that *b* is on the order of µm and, thus, is significantly larger than *t* on the order of 0.1 µm [43, 44].

Figure 2c shows an optical image of the freestanding LSCF-SDC VAN film, while Fig. 2d and e show top-down SEM images of the film after transferring to a glass substrate. Furthermore, Fig. 2f shows a cross-sectional SEM of the freestanding film. Together, Fig. 2d–f confirm the macroscopically defect free nature of the films. The films show no cracks and are readily transferrable, thus highlighting the exceptional mechanical properties of the VAN structure [3].

### Compositions of planar and VAN films explored in this work (differing levels of lattice mismatch strain with substrate)

To better understand how strain between the film and the substrate influences the delamination process, we now explore the structures and strain states (before and after lift-off) for each of the six film compositions under study (shown in Fig. 1), with each film grown on single crystal NaCl, to a thickness of ~ 225 nm. The lattice parameters of these different materials at 20 °C and 590 °C, and the strain levels with respect to the NaCl are shown in Table 1. It is observed that at 590 °C the lattice parameters of all the films are lower than those of the NaCl, hence promoting a negative lattice mismatch strain.

**Table 1:** Summary of lattice parameters for NaCl, SDC, LSCF, and BTO at 20 and 590 °C, as well as % difference to NaCl lattice parameter

Material	Lattice Parameter at 20 °C (Å) / % difference to NaCl	Lattice Parameter at 590 °C (Å) / % difference to NaCl	Change in lattice parameter between 590 °C and 20 °C (Å)/ % change
SDC [45]	5.43/− 3.55%	5.51/− 5.00%	− 0.08/− 1.45%
YSZ [46]	5.13/− 8.88%	5.17/− 10.90%	− 0.04/− 0.77%
LSCF [47]	*5.50/− 2.31%	*5.54/− 4.48%	− 0.04/− 0.72%
BTO [48]	*5.70/ + 1.24%	*5.77/− 0.52%	− 0.07/− 1.21%
STO [49]	*5.52/− 1.91%	*5.55/− 4.3%	− 0.03/− 0.54%
YSZ-STO**	5.33/− 5.40%	5.36/− 7.60%	− 0.03/− 0.56%
LSCF-SDC**	5.47/− 2.84%	5.53/− 4.66%	− 0.06/− 1.08%
BTO-SDC**	5.57/− 1.78%	5.64/− 2.76%	− 0.07/− 1.24%
NaCl substrate [50]	5.63/0%	5.80/0%	− 0.17/− 2.93%

^*^These values denote equivalent lattice parameters of LSCF, BTO and STO upon 45° in plane rotation

^**^The average lattice parameter is quoted, although the true values likely differ as the film strain state is in fact dominated by the matrix perovskite phase (as the non-perovskite SDC phase pillars are isolated from the interface with the substrate) [30, 31]

Unlike the lattice parameters, the thermal expansion coefficients of all the film materials are very close at 9–13 × 10^–6^ [45, 47, 48, 51–54], around a factor of 4 lower than the NaCl value of 40 × 10^–6^ [50, 55]. Hence all the films, whether planar or VAN, will undergo a similar degree of compressive thermal expansion mismatch forces induced by the NaCl substrates upon cooling.

### Strain states in VAN films before and after lift-off: retention of vertical strain state

**Fig. 3:**
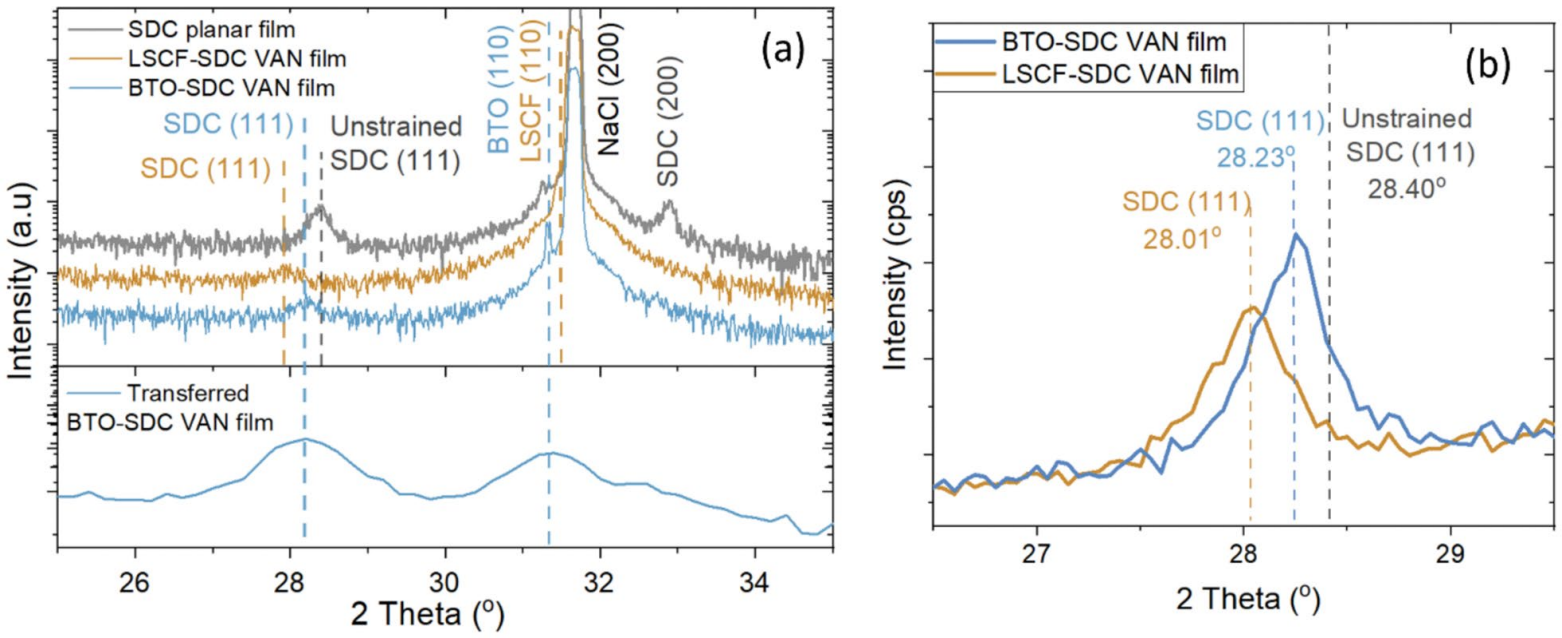
**a** XRD of planar SDC and LSCF-SDC and BTO-SDC VAN films grown on NaCl; [bottom] Grazing Incidence XRD of transferred BTO-SDC film showing strain state of SDC is retained after transfer **b** high-resolution SDC (111) peak for LSCF-SDC and BTO-SDC VAN films on NaCl

Figure 3 focuses in more depth on some of the data given in Fig. 1, showing the out-of-plane strain in planar films compared to VAN films. It thus compares the films containing SDC, either as single-phase planar films or SDC-containing VAN films.

We observe that the planar SDC film shows peaks corresponding to both (111) and (200) orientations, whereas the VAN films show only the (111) orientation. This single (111) SDC orientation in the VAN film is consistent with previous works on another SDC-perovskite VAN composition (SDC-SrTiO_3_ [56, 57]) where it is shown that the perovskite film matrix controls the orientation of the SDC nanopillars via vertical epitaxy.

The observed close positions of the BTO (110) and LSCF (110) peaks in the VAN films in Fig. 3 with the NaCl (200) substrate peak are indicative of highly oriented growth of the perovskite matrix materials on the NaCl. To confirm the shoulders on NaCl peaks are indeed (110) peaks corresponding to the perovskite film, 2θ-ω XRD scans were performed on the BTO-SDC VAN film using two different detectors, each equipped with a monochromator, in addition to scans of a pristine substrate. The results of these measurements are presented in Fig. S3. We observe separate and clear reflections distinct from the NaCl (200) peak which are not present when measuring the pristine substrate. Thus, we confirm the peak at 31.3° in the BTO-SDC VAN film originates from the BTO (110) reflections. The highly oriented nature of the perovskite peaks is further demonstrated from an X-ray Phi scan of an LSCF planar film on NaCl. The Phi scan of the (222) LSCF peak is shown Fig. S4, where four peaks of the LSCF rotated 45° with respect to the substrate confirm the structural relationship.

The positions of the (110) peaks in the BTO and LSCF planar films (2q = 31.51° and 31.37°, respectively) are shifted to lower angles than would be expected for the unstrained planar films, indicating an expansion in the out-of-plane lattice parameter. This is consistent with thermal contraction of the NaCl substrate of − 2.93% (from 5.8 Å to 5.63 Å) upon cooling to room temperature. To the best of our knowledge, this work therefore marks the first time highly oriented perovskite oxide thin films have been grown on NaCl substrates, and this is crucial to enabling the deposition of VAN films.

In VAN films, the unusual vertical control of strain state from the vertical heteroepitaxy between matrix and pillars is well known [18, 34, 58]. We note here that the close overlap of the perovskite (110) and NaCl (200) peaks prohibits a precise calculation of the strain levels along the vertical interfaces. Nevertheless, the matrix control of the strain state of the SDC is confirmed by the SDC (111) peaks, being in different positions for the two perovskite materials. As shown in Fig. 3b, 2θ_SDC(111)_ = 28.01° for the LSCF-SDC VAN and 28.23° for the BTO-SDC VAN. These values compare to 2θ_SDC(111)_ = 28.40° in the planar film. This is further confirmed by grazing incidence XRD scans (Fig. 3a–bottom panel) of the BTO-SDC VAN film before and after transfer to an arbitrary glass substrate. We observe that the BTO (110) and SDC (111) peaks are in the *same* positions before and after the lift-off and transfer. Hence, the strain state of the SDC is retained after the lift-off and transfer process, further proving that the substrate does not control the strain state of the VAN films, rather that the vertical relationship with the BTO in the VAN film does.

One may also consider cationic intermixing as a source of deformation of the crystal lattice [59]. However, in the example case of BTO-SDC, the ionic radius of Ti^4+^ (74 pm) is smaller than that of Ce^4+^ (97 pm), and thus Ti^4+^ substitution on the Ce^4+^ would result in a contraction of the SDC lattice parameter, rather than the expansion observed by XRD. The cell deformation is therefore attributed principally to strain in the nanocomposite structure.

In YSZ-STO nanocomposite films, no clear peaks from YSZ or STO were observed in the broad X-ray scan of Fig. 1q. However, a narrower scan (Fig. S5(a)) reveals the presence of the STO (110) peak close to the NaCl (200) peak. Also, a reciprocal space map (RSM) around the STO(103) is shown in Fig. S5(b). Here, clear peaks of both STO(103) and YSZ(114) are present, confirming highly oriented growth of these phases. The fact this is achieved despite the relatively low deposition temperature of 590 °C (epitaxial YSZ-STO films reported in previous literature have been deposited at 800 °C [56]), highlights the broad applicability of this film transfer strategy to a wide range of nanocomposite films. We note here the relatively broad nature of both peaks, which is consistent with the broad NaCl(204) substrate peak.

### Microstructures of VAN films: observation of fine columns and clear phase separation

**Fig. 4:**
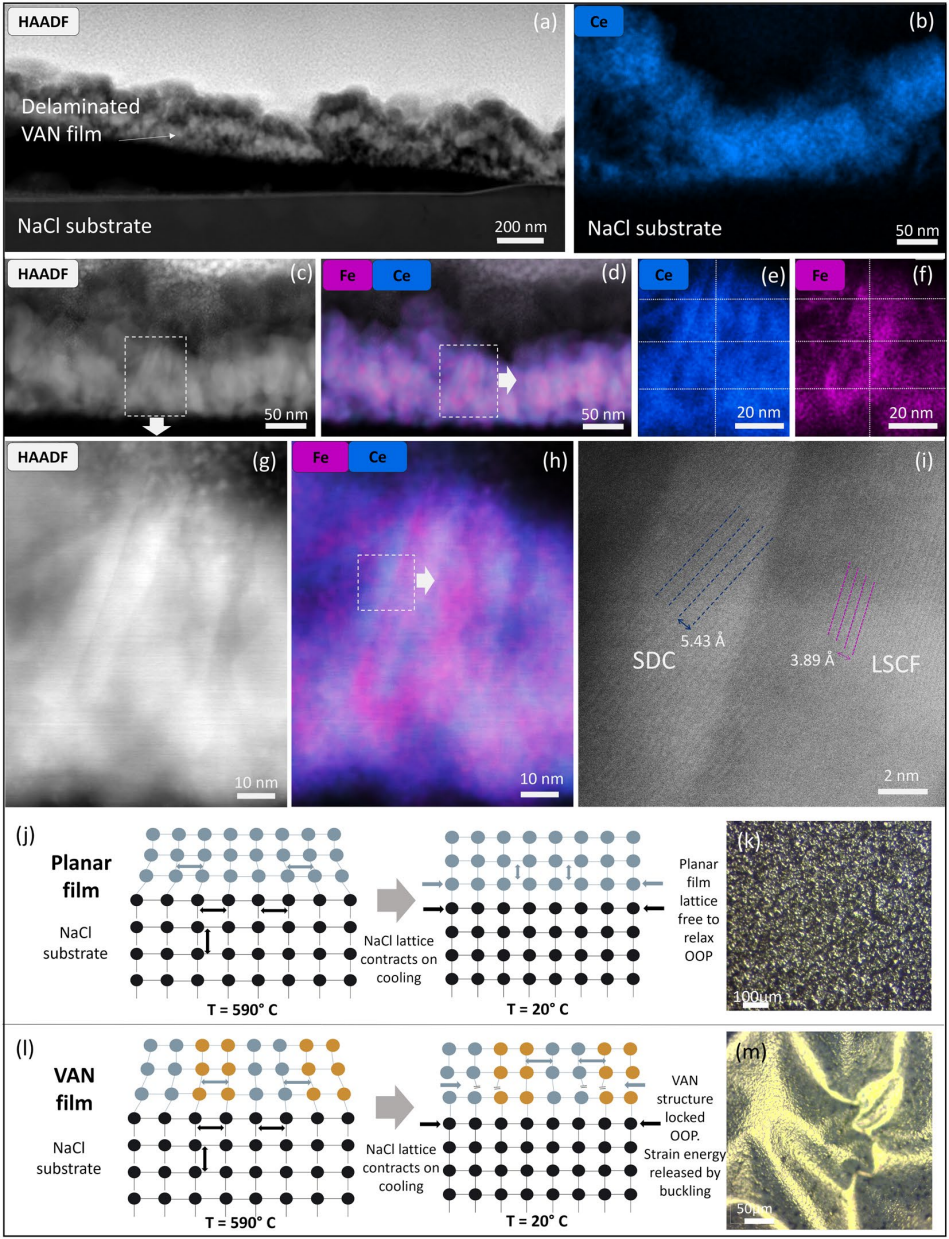
**a** HAADF-STEM image showing LSCF-SDC VAN films delaminated from NaCl substrate; **b** Ce EDX map showing ~ 15 nm thick film with a central region bonded to the substrate after buckling; **c** HAADF-STEM image showing freestanding region of LSCF-SDC VAN film; **d**–**f** EDX mapping showing nanostructure, **e** and **f** show region highlighted in (**d**), with gridlines added as a guide to the eye to emphasise separation between the Ce and Fe regions; **g** HAADF-STEM image of LSCF-SDC VAN film showing region highlighted in **c**; **h**) EDX map from region highlighted in (**c**) revealing separate Ce and Fe regions in the nanocomposite structure; **i** high-resolution STEM of region highlighted in (**h**) showing the crystalline structures of the SDC and LSCF columnar regions; **j** Schematic showing evolution of planar film-substrate lattice relationship upon cooling from the film growth temperature to room temperature; **k** Optical microscopy image of 225 nm thick SDC film on NaCl (image same as in Fig. 1d; **l** Schematic showing evolution of VAN film-substrate lattice relationship upon cooling from the film growth temperature to room temperature; **m** Optical microscopy image of 225 nm thick LSCF/ SDC VAN film on NaCl (image same as in Fig. 1j

Cross-sectional STEM images and EDX elemental maps of LSCF-SDC VAN films shown in Fig. 4 enable further insight into the structural nature of the nanocomposite films. The HAADF-STEM image in Fig. 4a confirms a clean interface where the film is delaminated from the NaCl substrate. We also observe that, where the film delaminates from the substrate, a thin (~ 15 nm) layer of the film remains bonded to the substrate, this is confirmed by EDX mapping (Fig. 4b).

Figure 4c–f demonstrate the preservation of the columnar nanocomposite structure, and separation of the LSCF and SDC phases, after the lift-off process: this is shown by the HAADF-STEM image (Fig. 4c) together with corresponding EDX elemental maps given in Fig. 4d, with Fig. 4e, f detailing the area highlighted by the dashed box in Fig. 4d. The preservation of the columnar nanocomposite structure in the delaminated film is further shown by the high-resolution HAADF-STEM image in Fig. 4g, acquired from the region indicated with a dashed box in Fig. 4c. The corresponding Fe and Ce EDX map in Fig. 4h reveals the formation of Fe and Ce-rich columns attributed to the LSCF and SDC phases, respectively, although the 3D nature of this nanostructure to some extent leads to overlap between Fe and Ce signals. We therefore further investigate this phase separation by acquiring atomic-resolution STEM images from the columns shown in Fig. 4g. Figure 4i confirms two adjacent columns with two different crystal orientations and lattice parameters, corresponding to LSCF and SDC phases. The phase separation observed here is consistent with the XRD results of Figs. 1 and 3, where distinct peaks for LSCF and SDC are clearly visible.

### Understanding why VAN films buckle and lift-off while planar films do not

The question remains as to why the single-phase planar films do not buckle and easily electrostatically lift-off, whereas the VAN films do. Below, we consider the influence of thermal expansion mismatch between the films and substrate, as well as defect levels, substrate surface instabilities, and Poisson ratios. Then, we propose a model to explain why the VAN films easily lift off, consistent with the different strain states in the VAN films compared to standard planar films (Fig. 4).

- a) Role of thermal expansion mismatch

For standard planar films, upon cooling from the growth temperature, the large in-plane compressive thermal expansion mismatch of the films and NaCl substrate (~ 1 × 10^–6^ cf. ~ 4 × 10^–6^) can be compensated by an out-of-plane tension, and hence expansion of the film out-of-plane, as schematically illustrated in Fig. 4j and from the XRD data of Fig. 1b and e of the standard planar LCSF and BTO films respectively. The optical micrograph of the non-buckled film is shown in Fig. 4k. We note that the film is cooled at a controlled at rate of ~ 10 °C/min, meaning there is sufficient time for the lattice to expand out-of-plane without inducing crack formation.

By contrast, for the VAN films one may first consider that the out-of-plane expansion is restricted, with the out-of-plane lattice parameters locked by the strain-coupled vertical interfaces which form between the two phases (Fig. 4l) [35, 58]. Moreover, as the same buckling is observed on used substrates (Fig. S6(c) and (d)), the buckling cannot be attributed to the nature of the bonding between the film and substrate, and must instead be attributed to the intrinsic nature of the nanocomposite films when placed under in-plane compression by the high thermal expansion coefficient NaCl substrate.

From Fig. 4a, b, it was noted that a thin (~ 15 nm) oxide layer remains bonded to the substrate even after buckling. It has been shown previously in nanocomposite thin films that vertical strain between the two nanocomposite phases becomes dominant over substrate-induced strain for film thicknesses above ~ 20 nm [60]. Thus, it can be understood that the initial ~ 15 nm of the VAN film is bonded to the substrate as the strain with the substrate dominates, at higher thicknesses the vertical strain locking between the two phases in the VAN films restricts the out-of-plane expansion, as confirmed in Fig. 3a.

We note also that, should there be any tensile strain from the macroscopic wrinkles, these have a large radius of curvature which minimises tensile stress concentration regions. Furthermore, if any tensile stress regions are present, as shown in a previous study for chemically lifted-off VAN films, the nanopillars act as crack blocking regions, leading to a higher fracture energy than in planar films [3].

- b) Role of defect levels in VAN films vs. planar films

It is also worth noting the potential role of defects within the nanocomposite structure contributing to the observed delamination. At the vertical interfaces between the two phases in the VAN films there will be a high defect density from lattice and structural mismatch effects. However, across the whole VAN film, lower defect densities typically result than in the planar films, owing to there being strain uniformity throughout the thickness in VAN films, which is not the case in planar films where there is gradual relaxation [58]. However, it is important also to consider the defects at the film/ substrate interface. The defects at this interface are again dominated by lattice and structural mismatch effects. For both the VAN and planar films, domain matching epitaxy (with associated dislocations) occurs [35], owing to the crystal structures of the phases in the films (both planar and VAN) being different to the rock salt structure of the NaCl. Hence, one could expect similar interfacial defect densities for both film types and thus no major difference in ease of delamination. Of course, we are here considering ‘conventional’ oxide epitaxy, and more work is now needed to understand precisely how oxide films (planar and VAN) grow on highly dissimilar crystals such as NaCl, and their associated defect landscapes.

- c) Role of structural instabilities of the NaCl substrate surface

One may also consider that structural instabilities of the NaCl substrates under the deposition conditions could contribute to the film detachment. AFM micrographs presented in Fig. S6 reveal significant changes in the NaCl surface microstructure before and after exposure to the deposition conditions (without film deposition), characterised by a decrease in surface roughness from ~ 11.7 nm to ~ 2.3 nm. However, as shown in Fig. S6, the same detachment of the nanocomposite films can be observed on both pristine and used substrates. Therefore, it may be concluded that the structural instability of the substrate does not play a significant role in the detachment of the films.

- d) Role of poisson ratio

Literature values of Poisson ratios of the materials under study in the VAN films are 0.29 for YSZ [61], 0.24 for STO [62], 0.32 for LSCF [63], 0.35 for BTO [64], and up to 0.56 in doped CeO_2_ [65]. Since all the VAN films readily lift-off regardless of these widely varying Poisson ratio mismatch between the films and the substrate (e.g., ~ 21% in YSZ-STO to ~ 75% in LSCF-SDC), this indicates it does not play a dominant role in the lift-off process.

The consideration of defects, structural instabilities, and Poisson ratios above indicates that these do not play determinant roles in the film lift off. On the other hand, we have shown that the large CTE mismatch between film and substrate combined with the unique strain states in the VAN films leads to the buckling. Thus in VAN films, the vertical strain independent of Poisson ratio effects [66, 67]. As the strain states are established during film growth, they remain ‘locked’ upon cooling as the pillars in the VAN films are not bound or strained by the substrate, but rather are embedded as a scaffold -type structure within the film. We also note that it is not necessary for thin films to exhibit chemical or structural perfection in order for strain states to significantly impact the physical properties of thin films, as shown in previous literature studies of polycrystalline thin films [68–70].

The buckling and lift-off process demonstrated in this work is not observed when growing VAN films on standard perovskite single crystal substrates [23, 35, 60, 71]. This is understood based on the fact that there is not the large CTE mismatch on the isostructural perovskite substrate.

### Physical properties of lifted-off VAN films compared to same films grown on single crystal substrates

**Fig. 5:**
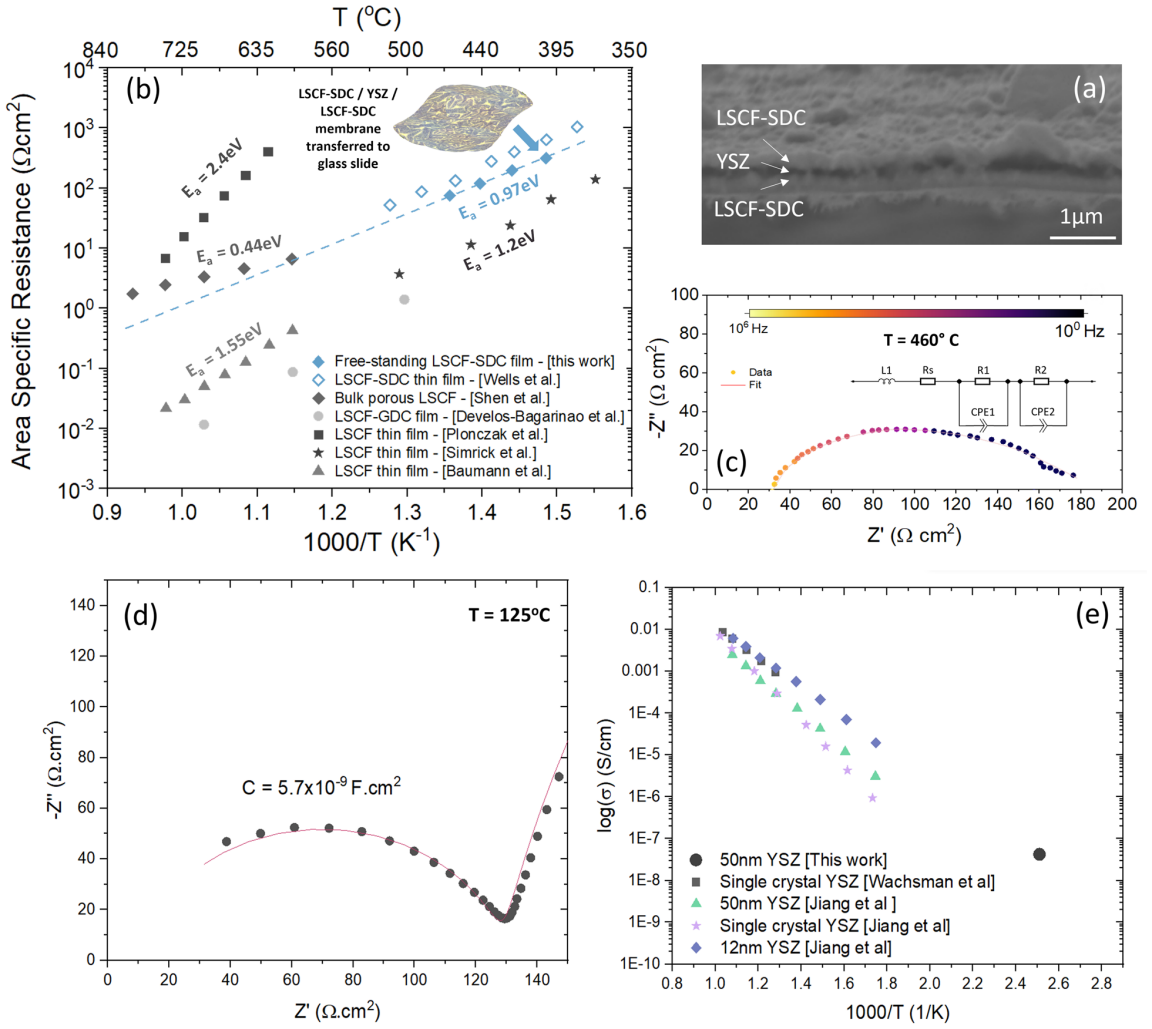
**a** Cross-sectional SEM of freestanding LSCF-SDC/YSZ/LSCF-SDC membrane **b** Arrhenius plots comparing area specific resistance (ASR) of LSCF-SDC/YSZ/LSCF-SDC freestanding SOC membranes with previous literature results for dense thin film and porous bulk LSCF [72, 73]; **c** representative Nyquist plot of the transferred LSCF-SDC/YSZ/LSCF-SDC membrane measured at 460 °C [inset: equivalent circuit model used to fit the data]; **d** Low-temperature Nyquist plot showing YSZ electrolyte impedance arc; **e** comparison of YSZ conductivity value with literature

Having demonstrated the successful transfer technique, it is important to prove that films lifted-off in this way have excellent physical performance and are not destroyed in any way by removal from the substrate. Thus, we now turn attention to the deposition and transfer of a complete mSOC membrane stack and compare performance to a typical, non-lifted-off, LSCF film grown on a standard ionic conductor substrate, as well as an equivalent bulk material. For the mSOC membrane stack, a ~ 225 nm thick LSCF-SDC VAN layer was deposited as before on the NaCl substrate. Subsequent layers of ionically conducting (Y_2_O_3_)_0.08_(ZrO_2_)_0.92_ (YSZ) (~ 100 nm) electrolyte and a further LSCF-SDC (~ 225 nm) cathode layer were deposited to create a proof-of-concept symmetric mSOC cell structure with a total membrane thickness of ~ 550 nm. The increased film thickness (compared to the previously studied ~ 225 nm single layer LSCF-SDC film) mitigated the need for a static charge to remove the film. Instead, the film was readily lifted from the substrate using a pair of tweezers. Figure 5a shows a cross-sectional SEM image of the multilayer mSOC membrane structure in which a clear distinction between the three layers is observed.

To confirm that the transfer process does not adversely affect the functionality of the VAN films, we transferred the LSCF-SDC/YSZ/LSCF-SDC membrane onto a porous Au paste current collector mounted on a glass slide and characterised the membrane by electrochemical impedance spectroscopy (EIS). The corresponding results are summarised in Fig. 5. Figure 5b shows the polarisation area specific resistance (ASR) of the freestanding LSCF-SDC/YSZ/LSCF-SDC membrane compared to dense LSCF thin films, LSFC-SDC and LSCF-GDC nanocomposite films, and bulk porous LSCF [26, 72–75]. Measurements were conducted in the 400–500 °C temperature range to align with previous measurements in literature of films with the same LSCF-SDC composition grown epitaxially on single crystal YSZ, and representative of targeted low-temperature SOFC application window. Here, the transferred membrane exhibits ASR values as low as 37 Ωcm^2^ at 500 °C and shows close comparison to literature values for the same LSCF-SDC composition grown epitaxially on single crystal YSZ. Literature values for the ASR of planar LSCF thin films are subject to wide variation. Nevertheless, due to a low activation energy of ~ 0.97 eV, the LSCF-SDC nanocomposite films of the present work can be considered to exhibit good electrochemical performance, particularly in low temperature regime, while extrapolation to higher temperatures reveals ASR values comparable to porous bulk LSCF. Hence, it can be concluded that, after lift-off and transfer, the films are of high quality without any macro-cracks or other defects that could obstruct the ionic current.

Figure 5c shows a representative Nyquist plot of the transferred LSCF-SDC/YSZ/LSCF-SDC membrane together with the equivalent circuit model, where L_1_ and R_s_ define the series inductance and resistance of the experimental setup, while R_1_, CPE_1_, R_2_ and CPE_2_ describe the polarisation resistance and parallel area specific capacitance respectively of the two LSCF-SDC electrodes, as typical of symmetric cell measurements [76]. The impedance response agrees with results previously observed for comparable VAN films [27–29]. To confirm the absence of electronic leakage through the 50-nm thick electrolyte, impedance measurements were performed at 125 °C to isolate the electrolyte impedance. As shown in Fig. 5d, the low temperature measurement reveals the presence of an impedance arc characterised by a capacitance value of ~ 5.7 × 10^–9^ F.cm^−2^ and a conductivity of ~ 4 × 10^–8^ S.cm^−1^. This impedance is attributed to the ionic conductivity of the electrolyte layer as the value agrees with literature values for bulk and thin film YSZ, as shown in Fig. 5e [77, 78].

### Surface chemical analysis of lifted-off VAN film by XPS

The key advantage of the work is the low-cost of the NaCl, the relative simplicity of the removal process, and the potential for flexible electronics, mSOCs, and possibly bioelectronic devices. However, we also need to consider the impact of possible Na and Cl contamination from the NaCl substrate. To do this, XPS measurements were performed on the LSCF-SDC films on the side of the films which was bonded to the NaCl substrate before being lifted off. The XPS-obtained elemental compositions, given in Table 2, are the mean values taken from five measurements at 2 different locations on the substrate/film interfacial surface. Excluding the C contribution from the analysis, we find that this interface contains 1.5 at.% Na. The consistency between results in different measurement locations indicates a high level of interfacial homogeneity.

**Table 2:** Elemental composition of transferred LSCF-SDC VAN films, measured at 2 regions on the side initially interfaced with the NaCl substrate

Element	Region 1 at.% (Excluding C)	Region 2 at.% (Excluding C)
C	–	–
La	4.2	4.0
Sr	5.2	5.1
Co	1.3	1.1
Fe	14.0	14.3
O	56.1	56.0
Sm	3.0	3.3
Ce	13.6	13.7
Na	1.5	1.6
Cl	1.1	0.9

We have also further explored the presence of Na *inside* the sample by carrying out ultra-low energy (200 eV) *in-situ* Ar surface cleaning to remove the native surface layer. At this depth level (a few monolayers from the substrate/film interface into the film), Na was < 0.4 at%. While these values are high enough to be detrimental to some technological applications, we highlight that XPS is a highly surface sensitive technique, and one would naturally expect some contamination from the part of the film that is in direct contact with the NaCl substrate. Complete XPS spectra are given in Fig. S7.

## Conclusions

In this work we introduce a novel strategy for achieving high quality lift-off and transfer of highly oriented oxide films in the form of vertically aligned nanocomposites (VANs). Based on the present study of 3 planar and 3 VAN films, the lift-off mechanism is independent of defect levels, Poisson ratios, or substrate surface instabilities, and is consistent with the large thermal expansion mismatch difference between the NaCl substrates and the VAN films, which results in large in-plane compressive stresses. In planar films, such stresses are compensated by an out-of-plane lattice expansion. By contrast, due to the unique strain states formed in VAN films, the in-plane compressive stresses cannot be relieved by a resultant out-of-plane tension the VAN films and is therefore released by buckling of the films.

Once buckled, the films are readily removed from the substrate by application of a small electrostatic force. Crucially, it is shown that such buckling does not lead to cracking in the VAN films owing to the fact the buckle radius is several orders of magnitude larger than the film thickness. Moreover, the VAN structures presented herein mark the first demonstration highly oriented perovskite oxide growth on NaCl substrates.

We prove the successful nature of our novel lift-off process (i.e. no structural or chemical damage), by the deposition and transfer of a freestanding 5 × 5 mm micro-solid oxide fuel cell membrane structure with thickness as low as ~ 550 nm. The membrane structure exhibits exceptional low-temperature electrochemical performance, characterised by area specific resistance (ASR) values of 37 Ωcm^2^ at 500 °C, comparable to equivalent VAN films grown epitaxially on single crystal YSZ. This work therefore marks an important step towards a new and simple oxide film lift-off strategy which avoids the possible degradation mechanisms of water-based dissolution methods. There is potential for widespread applicability of the method for making thin films not only for electrochemical devices, as demonstrated here, but also much more broadly in thin film oxide electronics where VAN films have a number of key advantages over standard planar films and are easy to grow [30].

## Experimental methods

Ceramic targets were prepared by grinding ~ 3 g powder for 30 min using an agate pestle and mortar. A 10-ton isostatic press was used to pelletised the powders, which were then sintered for 4 h at 1300 °C, with a heating/cooling rate of 5 °C/min. YSZ, SDC, LSCF and BTO targets were prepared using 3 g (Y_2_O_3_)_0.08_(ZrO_2_)_0.92_, (Sm_2_O_3_)_0.20_(CeO_2_)_0.80_, (La_0.60_Sr_0.40_)_0.95_Co_0.20_Fe_0.80_O_3,_ and BaTiO_3_ powder, respectively. LSCF-SDC and LSCF-BTO targets were made by mixing 1.5 g of the constituent powders. All powders were supplied by Alfa Aesar, with the exception of (La_0.60_Sr_0.40_)_0.95_Co_0.20_Fe_0.80_O_3_ supplied by Fuel cell materials, Nexceris, LLC.

Thin films of YSZ, SDC, LSCF, BTO, LSCF-SDC and BTO-SDC were grown by pulsed laser deposition (PLD) on (001) oriented single-crystal NaCl substrates (5 × 5 × 1 mm, Biotain Crystal Co., Limited). As NaCl is hygroscopic, the substrates were supplied individually sealed and were loaded into the deposition chamber without any further surface treatment. Substrates were first adhered to the heater via silver paste heated to 80 ℃ for 1 min with a ramp rate of 10 °C /min. Before each deposition, the chamber was evacuated to at least 8 × 10^–6^ mBar. The substrates were then heated to 590 °C. A 248-nm KrF laser (Lambda Physik, Inc) was used to ablate the ceramic targets, held 45 mm from the substrate, with a repetition rate of 3 Hz in an atmosphere of 0.4 mBar O_2_ with a gas flow rate of 6 sccm and a laser fluence of ~ 3.2 J/cm^2^ (a lower fluence of 1.6 J/cm^2^ was used for deposition of YSZ). After growth, samples were cooled at 10 °C/min to room temperature under the same 0.4 mBar O_2_ atmosphere as used during the deposition. Multilayer samples were deposited sequentially without breaking vacuum.

X-ray diffraction measurements were performed using a Panalytical Empyrean high resolution X-ray diffractometer (Cu-Kα radiation; λ = 1.5405 Å). Scanning electron microscopy images were acquired in Secondary Electron mode using a Nova NanoSEM 450 system. Atomic force microscopy measurements were performed with a Bruker Multimode 8 system operating in tapping mode. Silicon cantilevers (Budget Sensors Ltd; resonance frequency: 300 kHz; spring constant: 40 N/m) were used to image 0.5–1 μm^2^ areas at a scan frequency of 1 Hz. For electrochemical impedance spectroscopy measurements, a Materials Lab XM Impedance Analyser (Ametek) was used to acquire spectra over a frequency range 10^6^–1 Hz with a voltage amplitude of 10 mV. Measurements were conducted using porous Au paste current collectors applied to both sides of symmetric cell geometries, with samples mounted on a heating stage in a symmetric air atmosphere.

Room-temperature X-ray photoelectron spectroscopy (XPS) measurements were carried out in a Thermo Scientific Escalab 250Xi employing a monochromated Al k_α_ X-ray source (1486.7 eV). Different surface areas were analysed using a 650-µm^2^ X-ray beam, with a pass energy of 20 eV at a step size of 0.1 eV. Electronic charge neutralisation was obtained by employing an ion source. The spectra binding energies were calibrated based on the sample’s Fermi edge to avoid uncertainties arising from using the C 1 s peak from adventitious carbon.

High angle annular dark field STEM measurements were acquired using high resolution aberration correction Thermo Fisher Scientific Spectra 300, with a 300 keV, 100 pA electron beam and a dwell time of 500 µs. The convergence and collection angles were set to 24 mrad and 60–200 mrad respectively. EDX was collected by 4 Super-X EDX detectors through multiple frame acquastion with a total dwell time of. Samples were prepared using a FEI Helios Nanolab SEM/FIB for lamella preparation. EDX data were processed using Hyperspy [79]. To enable precise EDX mapping post processing frame-by-frame drift correction was implement before specific elemental EDX intensities.

## Supplementary Information

Additional file 1.

## Data Availability

The data that support the findings of this study are available in Figshare with the DOI https://doi.org/10.6084/m9.figshare.23627886
