# Supplementary material for: Novel electrostatic dry lift-off and transfer route for vertically aligned nanocomposite oxide thin films
Source: Nano Converg. 2025 Jul 18;12:37. doi: 10.1186/s40580-025-00494-1 (PMC12274158; doi:10.1186/s40580-025-00494-1)
Supplement: Supplementary file 1 — Additional file 1. [file 40580_2025_494_MOESM1_ESM.docx]

Novel electrostatic dry lift-off and transfer route for vertically aligned nanocomposite oxide thin films

- Supplementary Information

*Matthew P. Wells^*1^, Babak Bakhit^1,2^, Simon M. Fairclough^1^, Jordi J. H. Weingard^1^, Caterina Ducati^1^, Judith L. MacManus-Driscoll^1^*

^1^Department of Materials Science and Metallurgy, University of Cambridge, 27 Charles Babbage Road, Cambridge CB3 0FS, United Kingdom.

^2^Thin Film Physics Division, Department of Physics (IFM), Linköping University, Linköping SE-58183, Sweden


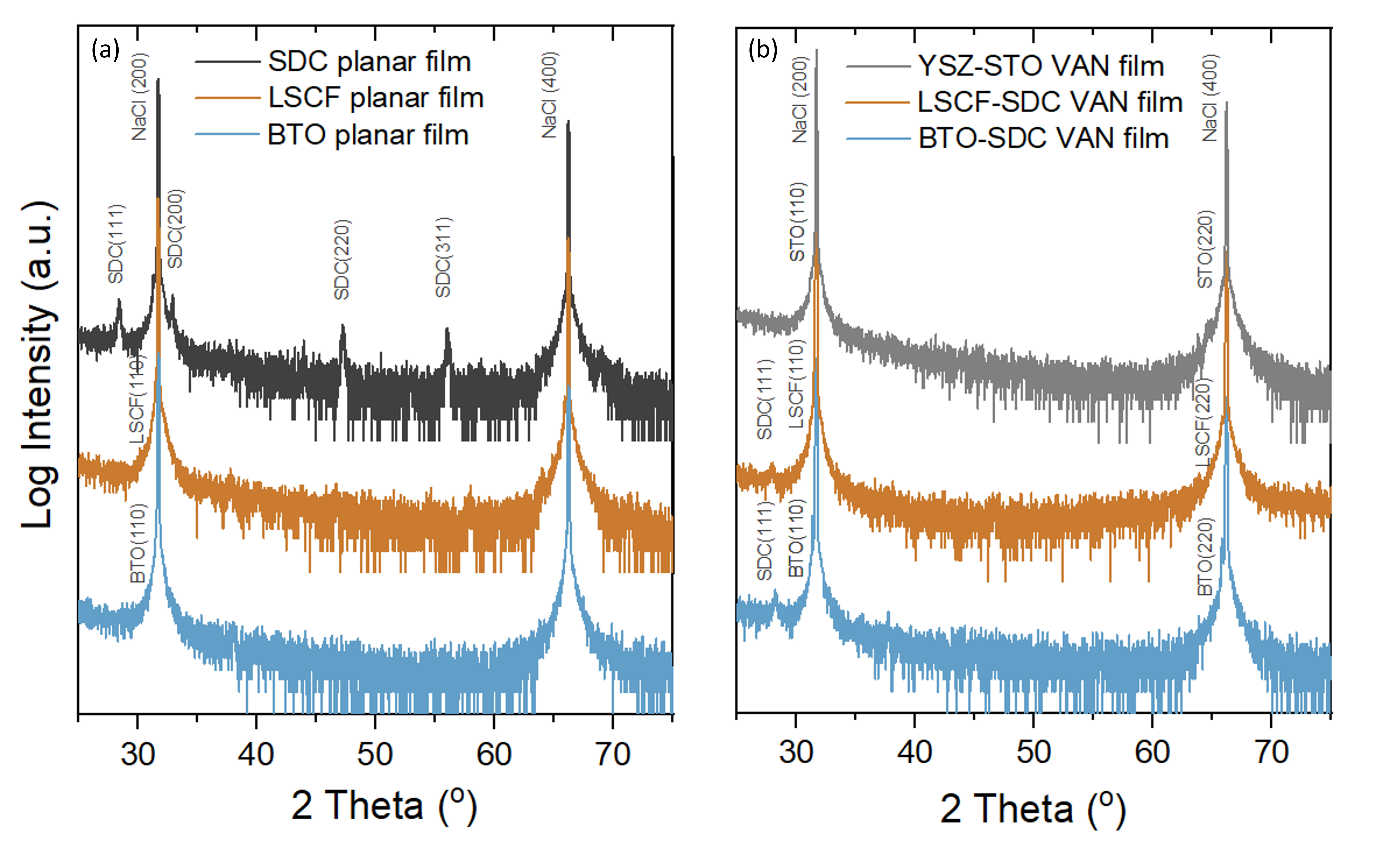


Figure S1: XRD plots for all a) planar and b) VAN films


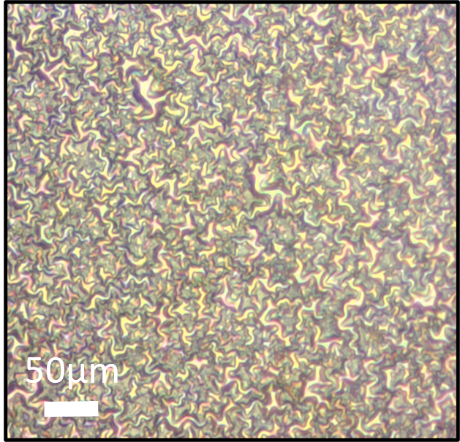


Figure S2: Optical microscopy image of 50nm thick LSCF-SDC VAN film


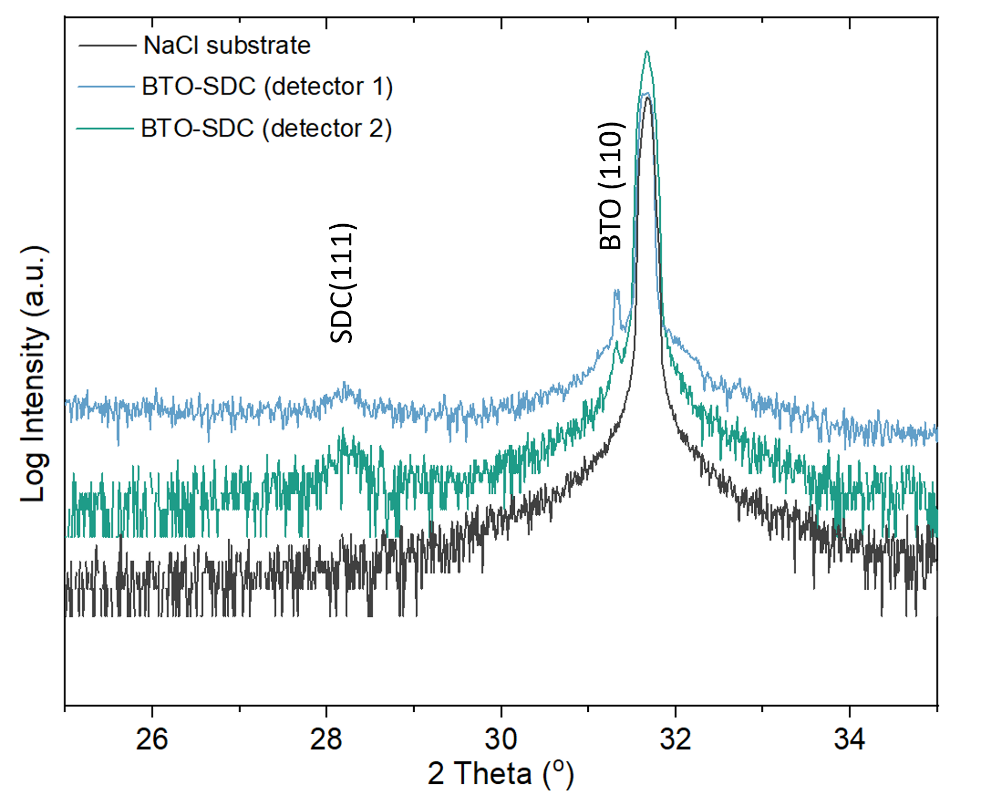


Figure S3: XRD of BTO-SDC VAN films together with uncoated NaCl substrate with data measured by two different detectors


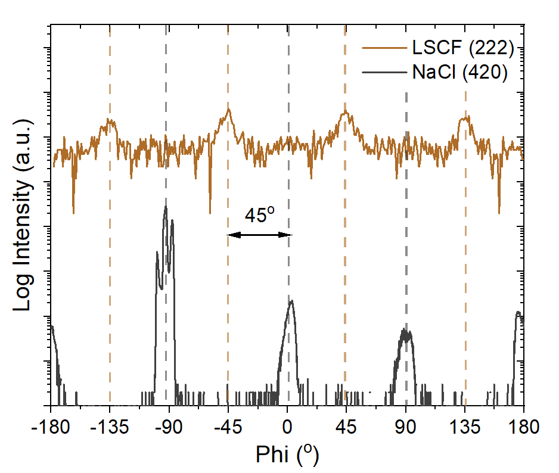


Figure S4: Phi scan around LSCF (222) and NaCl (420) peaks showing 45° rotational epitaxial relationship between film and substrate


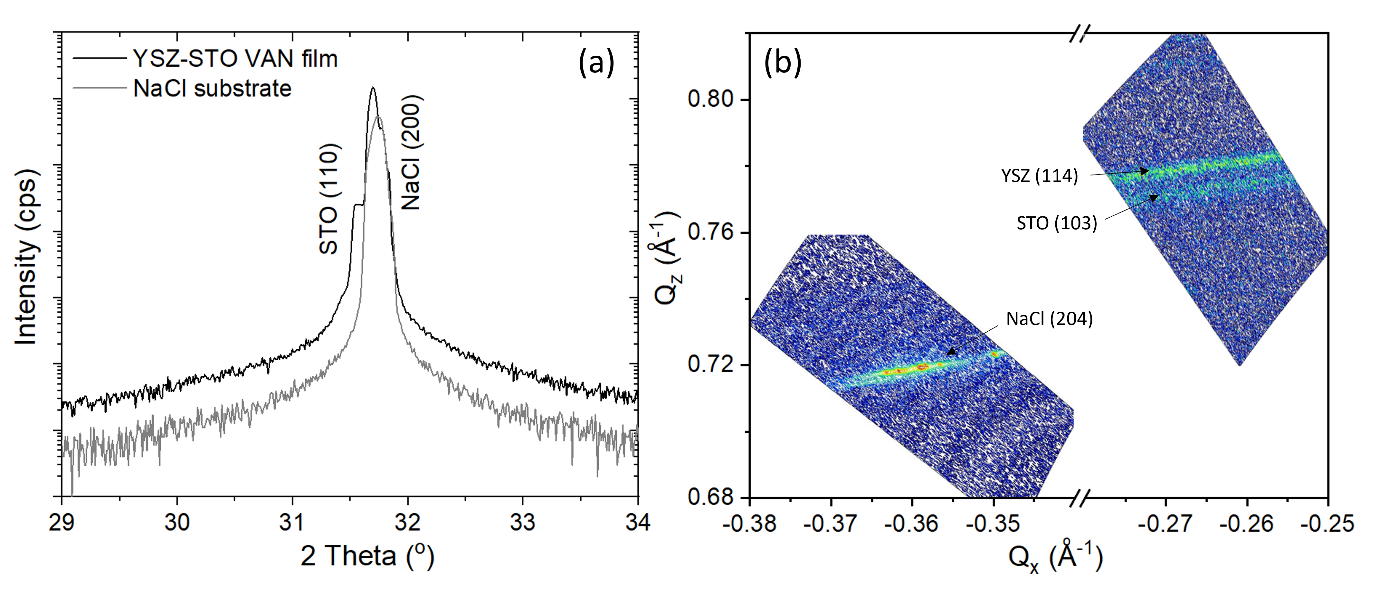


Figure S5: a) XRD plot of YSZ-STO VAN film together with uncoated NaCl substrate b) Reciprocal Space Map showing NaCl (204), STO (103) and YSZ (114) peaks


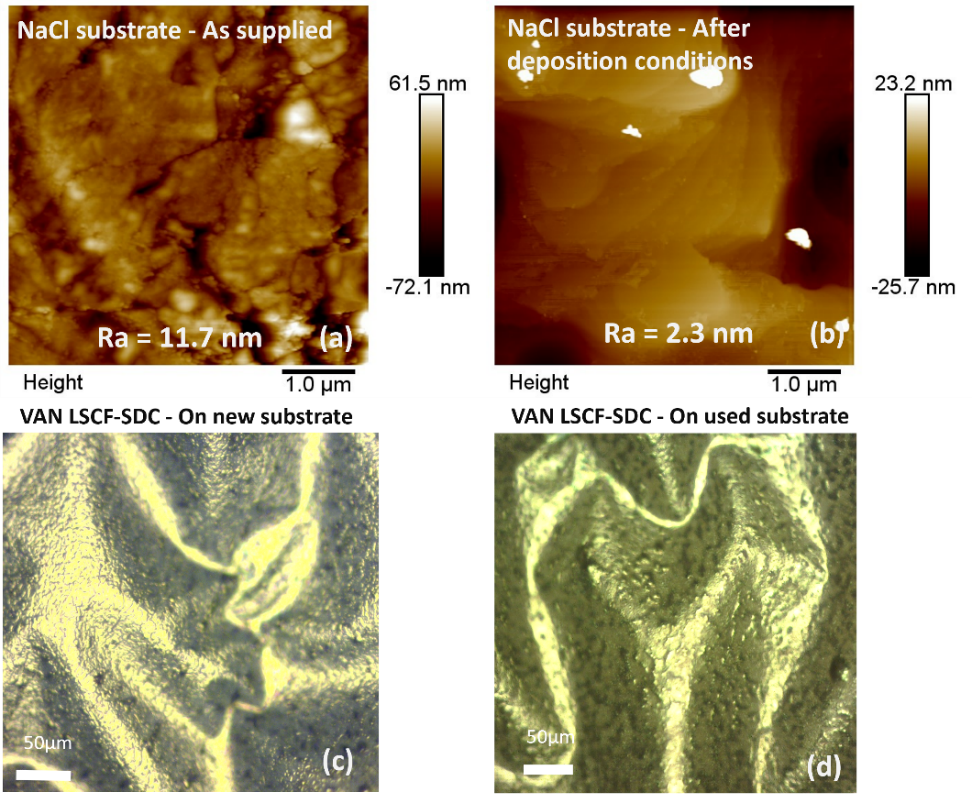


Figure S6: AFM micrographs of NaCl substrate surface a) before and b) after exposure to deposition conditions (590° C, 0.4 mBar O_2_); Optical microscopy images of LSCF-SDC VAN films grown on c) pristine and d) used NaCl substrates showing buckling occurs regardless of substrate microstructure.s


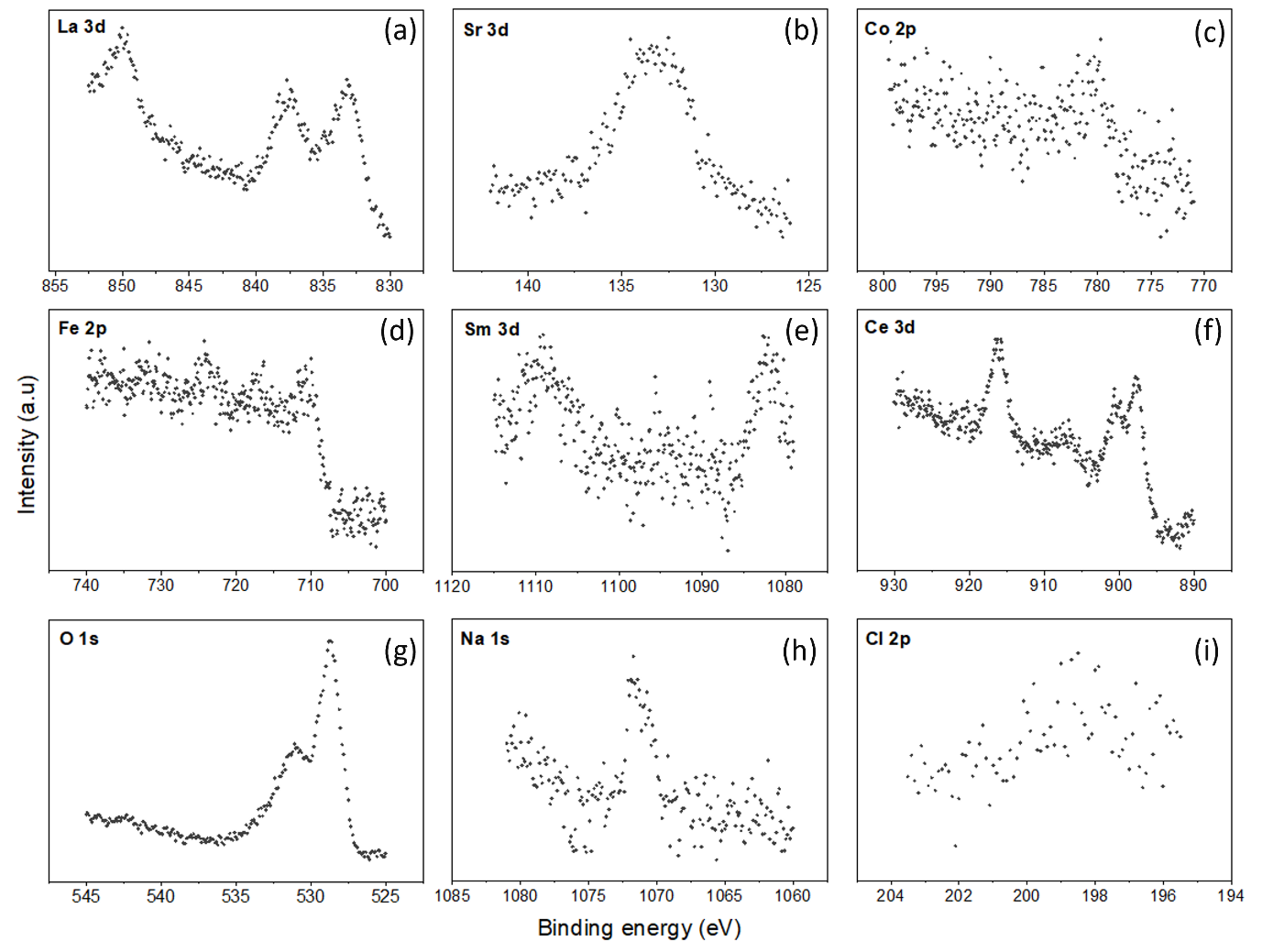


Figure S7: XPS spectra for: a) La 3d; b) Sr 3d; c) Co 2p; d) Fe 2p; e) Sm 3d; f) Ce 3d; g) O 1s; h) Na 1s; i) Cl 2p
